# Climate Change and Its Impacts on Farmer’s Livelihood in Different Physiographic Regions of the Trans-Boundary Koshi River Basin, Central Himalayas

**DOI:** 10.3390/ijerph18137142

**Published:** 2021-07-03

**Authors:** Basanta Paudel, Zhaofeng Wang, Yili Zhang, Mohan Kumar Rai, Pranesh Kumar Paul

**Affiliations:** 1 Key Laboratory of Land Surface Pattern and Simulation, Institute of Geographic Sciences and Natural Resources Research, Chinese Academy of Sciences, Beijing 100101, China; paudelb@igsnrr.ac.cn (B.P.); wangzf@igsnrr.ac.cn (Z.W.); mkrai2019@igsnrr.ac.cn (M.K.R.); 2 Kathmandu Center for Research and Education, Chinese Academy of Sciences–Tribhuvan University, Kathmandu 44613, Nepal; 3 Department of Land Change Science and Biogeography, University of Chinese Academy of Sciences, Beijing 100049, China; 4 Key Laboratory of Water Cycle and Related Processes, Institute of Geographic Sciences and Natural Resources Research, Chinese Academy of Sciences, Beijing 100101, China; praneshkp@igsnrr.ac.cn

**Keywords:** climate change, impact, adaptation strategies, livelihood, Koshi River Basin

## Abstract

The impact of climate change on farmers’ livelihoods has been observed in various forms at the local and regional scales. It is well known that the Himalayas region is affected by climate change, as reflected in the basic knowledge of farmers in the region. A questionnaire-based survey involving a total of 747 households was conducted to gather information on climate change and its impact, where the survey addressed four physiographic regions of the trans-boundary Koshi River Basin (KRB). Moreover, climatic data were used to calculate climatic trends between 1980 and 2018. The Mann–Kendall trend test was performed and the Sen’s slope calculated to analyze the inter-annual climatic trends over time. The survey noted that, for the basin, there was an increase in temperature, climate-induced diseases of crops, an increase in the frequency of pests as well as drought and floods and a decrease in rainfall, all which are strong indicators of climate change. It was perceived that these indicators had adverse impacts on crop production (89.4%), human health (82.5%), livestock (68.7%) and vegetation (52.1%). The observed climatic trends for all the physiographic regions included an increasing temperature trend and a decreasing rainfall trend. The rate of change varied according to each region, hence strongly supporting the farmers’ local knowledge of climate change. The highest increasing trend of temperature noted in the hill region at 0.0975 °C/a (*p* = 0.0002) and sharpest decreasing trend of rainfall in the mountain region by −10.424 mm/a (*p* = 0.016) between 1980 and 2018. Formulation of suitable adaptation strategies according to physiographic region can minimize the impact of climate change. New adaptation strategies proposed include the introduction of infrastructure for irrigation systems, the development of crop seeds that are more tolerant to drought, pests and disease tolerance, and the construction of local hospitals for the benefit of farming communities.

## 1. Introduction

At a global level, it has been widely recognized that for decades, there has been a long-term trend of increasing temperatures [1,2], and the long-term changing status of climatic variables show that the climate is changing [3]. Many studies point to the negative impacts of climate change on peoples’ livelihoods [4], farming sector [5,6], and on the crop-livestock systems [7]. The agricultural system, human settlement, and crop yield production are also highly affected by climate change in different parts of the globe [8,9,10]. The study showed that there was an influence of climate change on agriculture and resulted in a reduction of productivity up to 21% [11]. Thus, the climatic variability and its impact reduces the economic growth in many countries worldwide [12]. In the Himalayas region, the impact of climate change is widely perceived by farmers as negatively affecting the farming sector [13,14], and the farmers’ livelihoods [15]. A recent study in Nepal reported that the annual average temperature has risen by 0.05 °C/a between 2000 and 2015 and, at the same time, precipitation has decreased by −16.09 mm/year [16]. Furthermore, for the central Himalayas region, it was concluded that the increase in temperature and the decrease in precipitation negatively affected farm activities and the farmers’ livelihoods [17]. In this area, the majority of farmers grow crops that are heavily dependent on precipitation [18]. Frequent droughts and short-term heavy rainfall make conditions for farming problematic because such events tend to lower crop production [19].

The farmers’ basic knowledge on climatic events and the impact of climate change does have great significance in the context of formulating adaptation strategies to mitigate the overall impact of climate change [20]. Studies have pointed out that farmers have attempted to minimize the effects of climate change by applying their own knowledge and measures to crop plantation [21], change in crop types, and crop rotations [22,23]. A national level study in Nepal reported the introduction of crop varieties with mixed cropping, the use of chemical fertilizers, and the introduction of measures to control climate-induced diseases of crops and livestock in order to improve agricultural output [15]. Furthermore, based on the native people in different ecological regions, farmers have become more aware of possible changes of the impact of overall climatic conditions in their locality [24]; nevertheless, farmers may not be completely aware of the impact of climate change on their activities and livelihoods [16].

Previous climate change studies in different parts of the Himalayas region note that the increasing trends of temperature [25] and erratic rainfall [26] events frequently cause flood-related disasters [27]. Moreover, floods frequently occur in the trans-boundary Koshi River Basin (KRB) [28]. In 2008, the floods damaged many properties of downstream residents in the basin. The farmers’ livelihoods, in the downstream regions of the KRB, were highly affected by such events [29]. The majority of residents in the different parts of the Himalayas region are now aware of climate change, but their understanding of its impact vary depending on the different geographical locations and the climatic conditions [30]. It is clear that individuals engaged in agricultural activities have a high awareness of the small ups and downs of the climate change in their local topography [16,31]. Therefore, use of the farmers’ perception and their local knowledge is one of the best approaches for monitoring and dealing with the impact of climate change. Such an approach should prove invaluable when it comes to formulating adaptation strategies to minimize the impact of climate change [32]. Furthermore, a farmers’ perception-based survey supports understanding and knowledge sharing between farmers and policymakers to improve the existing adaptation strategies, plans, and policies [15].

The perception-based study of climate change has received much attention in recent times because it assists policymakers to build appropriate adaptation strategies to minimize the impact of climate change on the farmers’ livelihoods and on the farming sector in general. Due to the highly visible impacts of climate change over the past decade, the farmers’ daily life and overall livelihood is very vulnerable, particularly in the central Himalayas region and the trans-boundary KRB regions, where the majority of people depend mainly on farming activity. Furthermore, there is a need to examine and study farming using an integrated approach, which combines the observed climatic trends and agricultural data with the farmers’ perception of climate change and its impact on livelihoods. Additionally, this combined approach should help to improve existing adaptation strategies and allow us to formulate new adaptation strategies to minimize the impacts of climate change in the central Himalayas region. Although the impact of climate change on farmers’ livelihoods in the trans-boundary KRB is noticeable, studies examining the different physiographic regions of the basin are limited. Moreover, given the different climatic zones, the changing climatic conditions and the different physiographic regions, adaptation strategies implemented in the past may not be appropriate now in present times. Therefore, it is very relevant to undertake research concerning the farmers’ perception of the impact of climate change in the different physiographic regions to assess the farmers’ understanding of climate change. Thus, the aim of the present study was to address this research gap and assess the impact of climate change on farmers’ livelihoods in the four different physiographic regions of the trans-boundary KRB. This study deals with the KRB, which covers both the changing climatic trends between 1980 and 2018 in the four different physiographic regions (three in Nepal and one in India) and the farmers’ perception of it. We hope that the findings of this study will prove useful for developing suitable adaptation strategies that support minimization of the impact of climate change on farmers’ livelihoods in the Himalayas region and in other regions of the world with similar topography.

## 2. Materials and Methods

### 2.1. Study Area

**Figure 1:**
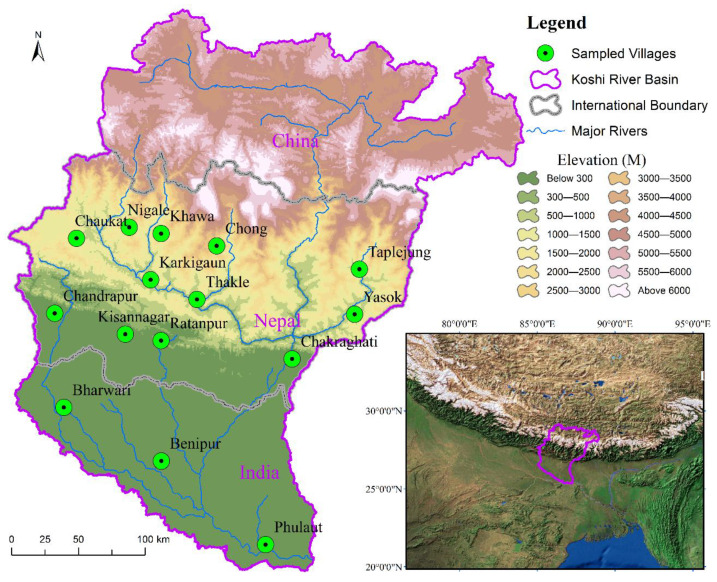
Map showing the sampled villages in the study.

The trans-boundary KRB region, covering an area of 87,500 km^2^, is located in the central Himalayas [33] (Figure 1). The study focused on the central (Nepal) and southern (India) parts of the trans-boundary KRB. In this study, we selected 15 different villages from four physiographical regions of the KRB. Out of them, four villages were located in a mountain region and another four in a hill region. Similarly, four villages were selected in the Tarai region and the remaining three villages were in the Gangetic plain (Table 1).

The elevation of the villages varied, being around 2200 m (above mean sea level: a.m.s.l.) for the mountain region and around 1200 m (a.m.s.l.) for the hill region. The villages in the Tarai region were located at about 100 m (a.m.s.l.) and around 50 m (a.m.s.l.) in the Gangetic plain, respectively. Due to the altitude and climatic variations of the villages, the crops also varied according to the physiographical region [34]. The main crops found in the mountain region were potatoes and maize; in the hill region, it was maize and millet; while rice, wheat, and maize were the main crops in the Tarai region and the Gangetic plain.

### 2.2. Study Area Data Collection

#### 2.2.1. Climatic Data and Survey Questionnaire

Climatic change cannot be deduced from short-term of data, since two periods of at least 30 years are required and when the World Meteorological Organization (WMO stipulates that calculation of “climate normals” requires “having data available in 24 or more out of the 30 years” [35]. Thus, the study used station-based climatic data (mean annual temperature and total annual rainfall; a total of 107 stations for rainfall and 53 stations for temperature) for the four physiographic regions (Nepal and India) of the KRB between 1980 and 2018. The climatic data for 27 stations (27 for rainfall and nine for temperature) for the mountain region, and 45 stations (45 for rainfall and 17 for temperature) for the hill region were used. Similarly, climatic data for 19 stations (19 for rainfall and 11 for temperature) from the Tarai region of Nepal were used with the data from these three regions (mountain, hill and Tarai) being collected by the Department of Hydrology and Meteorology of the Government of Nepal. In addition, climatic data for 16 stations in the Gangetic plain (Koshi River Basin, India), collected by the Indian Meteorology Department of the Government of India, were used in the study.

The farmers’ local knowledge is often used to evaluate the perception of climate change [36], and its impact [16]. Thus, a semi-structured questionnaire was developed to assess the farmers’ perception of climate change and its impact in the different physiographic regions of the KRB (Table 1).

This study first selected 15 different villages based on the spatial representation of each physiographic region, and a similar elevation range for each region with regard to a better understanding of climate change and its overall impact. In the second step, we pre-tested 30 household surveys (HSs) in two villages (Mude and Dhulikhel) of the KRB for clarity of the questionnaire and its understanding by respondents. During pre-test HSs, we noted that most respondents easily understood our questionnaire, and then we confirmed it to apply in this study. In the third step, a simple random sampling method was applied in conducting the HSs for each selected village. Furthermore, we performed over 10% HSs between 32 and 57 households within the selected villages based on total household numbers (Figure 1). Thus, a total of 747 HSs in 2018 and 2019 in 15 villages and covering the four physiographic regions of the trans-boundary KRB were undertaken.

#### 2.2.2. Interviewees and Focus Group Discussions

To acquire in-depth local knowledge regarding climate change and its impact on farmers, we selected six key interviewees (KII) from each village. The KII selected included the local leaders, the chairpersons of the villages, the chairpersons of the local farming groups, the lead persons for the local women groups, the chairpersons of the youth farming groups and/or the youth clubs, the chairpersons or members of the community forestry users groups, teachers, and leading farmers in the villages. Furthermore, to gain an overall perception of the residents’ views on climate change and its impact in each village, we also conducted focus group discussions (FGDs) in each village involving the aforementioned chairpersons/leaders. It became clear from the FGDs that there was a good overall appreciation of the effects of climate change in the specific regions and its impact on the farmers’ livelihoods in the various villages in the different physiographic regions of the KRB.

### 2.3. Data Analysis

This study utilized station-based daily climate data of temperature and rainfall during 1980–2018 for the different regions, and the climatic trends were calculated. For this, we performed a Mann–Kendall trend test [37,38] and calculated the Sen’s slope [39] to analyze the inter-annual climatic trends (annual mean temperature and total annual precipitation, respectively) between 1980 and 2018; also the significance of the variation (*p* value) was tested at the 95% confidence level. There has been wide use of the Mann–Kendall trend test and Sen’s slope for time series climatic data analysis [40]. The calculated and performed (Equations (1)–(7)) Mann–Kendall trend test statistics (S) and Sen’s slope was and defined as:

(1) $$S=\overset{n-1}{\underset{i=1}{\sum }}\overset{n}{\underset{j=i+1}{\sum }}sgn\left(X_{j}-X_{i}\right)$$

where *S* is the result of the sum of the counts of (*X_j_ − X_i_*); $X_{i}$ and $X_{j}$ are the time series data values [40]; *n* is the number of data points in the time series; and *sgn* (*X_j_ − X_i_*) is the sign function as:

(2) $$sgn\left(X_{j}-X_{i}\right)=\{\begin{array}{c} +1,\;\mathrm{if}\;X_{j}-X_{i}>0\\ 0,\;\mathrm{if}\;X_{j}-X_{i}=0\\ -1,\;\mathrm{if}\;X_{j}-X_{i}<0 \end{array}$$

The variance was calculated as:

(3) $$Var\left(S\right)=\frac{n\left(n-1\right)\;\left(2n+5\right)-\sum_{i=1}^{m}t_{i}\left(t_{i}-1\right)\;\left(2t_{i}+5\right)}{18}$$

where *n* is the number of data in the time series; m represents the number of tie groups; and *t_i_* indicates the number of ties of extent *i*. If the sample size is greater than 10, the test statistic *Z_S_* is calculated [40] utilizing Equation (4):

(4) $$Z_{s}\left(X_{j}-X_{i}\right)=\{\begin{array}{c} \frac{S-1}{\sqrt{Var\;\left(S\right)}},\;\mathrm{If}\;S>0\\ +0,\;\mathrm{if}\;S=0\\ \frac{S-1}{\sqrt{Var\;\left(S\right)}},\;\mathrm{If}\;S<0 \end{array}$$

The significant trend was evaluated using the *Z_s_* value. A positive value of *Z_s_* indicates an increasing trend and its negative value a descending trend. The level of significance for this study setup was 5% (α = 0.05). Furthermore, Sen’s slope was calculated utilizing Equations (5)–(7) developed by Sen (1968) as:

(5) $$Q_{i}=\frac{X_{j}-X_{k}}{j-k},\;\mathrm{for}\;i=1,\;\ldots \;\ldots \;\ldots ,N$$

where $X_{j}$ and $X_{k}$ are the data values at times *j* and *k* (*j* > *k*), respectively [40]; and *N* is the total sample number of observations. Furthermore, *N* values of $Q_{i}$ are arranged based on its value from lower to higher order and the median of thee slope or Sen’s slope calculated as:

(6) $$Q_{med}=\{\begin{array}{c} Q_{\left[\left(N+1\right)/2\right],}\;\mathrm{If}\;N\;is\;odd\\ \frac{Q_{\left[N/2\right],}+Q_{\left[\left(N+2\right)/2\right],}\;\mathrm{If}\;N\;is\;even}{2}\; \end{array}$$

The $Q_{med}$ is the reflection of the data trend, its value points out the steepness of the trend, and confidence interval was calculated based on Equation (7) as:

(7) $$C_{\alpha }=Z_{1-\frac{\alpha }{2}}\sqrt{Var\;\left(S\right)}$$

where $Z_{1-\alpha /2}$ indicates standard normal distribution and Var (S) is defined in Equation (3).

Furthermore, this study was also based on primary field survey data including socioeconomic data and local knowledge of the farmers concerning climate change and its impact. Thus, the observed indicators of climate change by local farmers’ in four different physiographic regions of the trans-boundary KRB were analyzed by each region. In addition, the observed adverse impacts of climate change by local respondents’ in the various sectors for the trans-boundary KRB were also performed by each physiographic region of the basin.

## 3. Results

### 3.1. Climatic Trends for the Different Physiographic Regions

**Figure 2:**
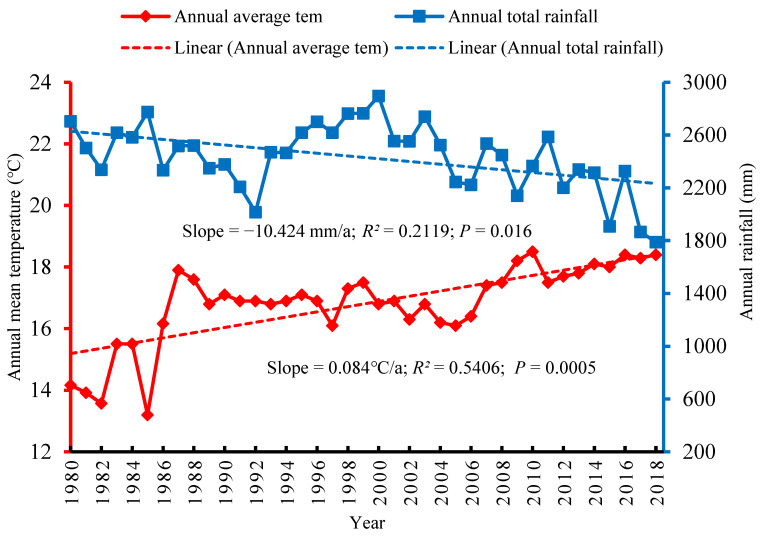
Climatic trend in the mountain region of the trans-boundary KRB.

The climatic trends in the mountain region of the KRB indicated that the mean annual temperature increased noticeably at a rate of 0.084 °C/a between 1980 and 2018. The increased rate of temperature positively correlated with the *p* value of 0.0005 at the 95% confidence level (Figure 2). At the same time, the total annual precipitation of the region exhibited a decreasing rate of precipitation of −10.424 mm/a. The total annual precipitation over the time period also positively correlated with a *p* value of 0.016. The climatic trend observations for the mountain region clearly indicated that climate change had impacted the region between 1980 and 2018 and thus there was also a high probability that this had impacted on agriculture and the farmers’ livelihoods.

**Figure 3:**
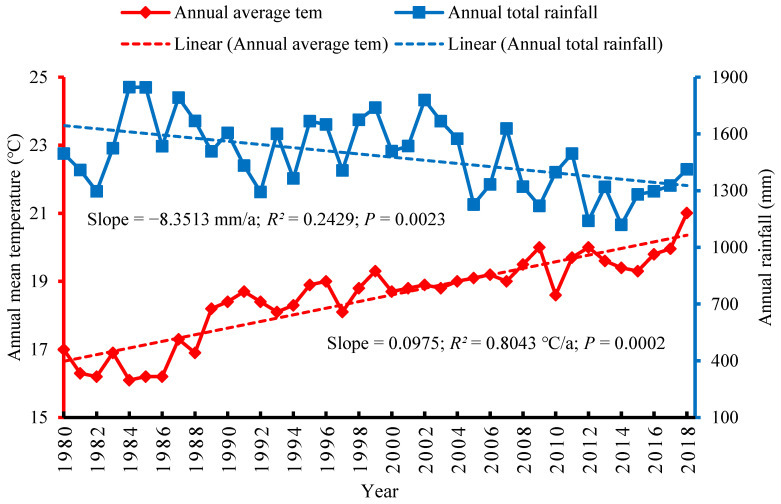
Climatic trends in the hill region of the trans-boundary KRB.

Climatic trends for the hill region of the basin indicated that the mean annual temperature had also risen significantly at a rate of 0.0975 °C/a between 1980 and 2018. The increased rate of temperature for this region was also positively correlated with a *p* value of 0.0002. The trend for the annual rate of temperature increase was highest for the hill region than other regions of the basin. Similarly, the total annual precipitation between 1980 and 2018 in the hill region showed a sharply decreasing trend of −8.3513 mm/a. The total annual precipitation for this region also positively correlated with a *p* value of 0.0023 (Figure 3). The station-based climatic trends for the hill region of the basin did indicate noticeable climate change scenarios over the period 1980 and 2018 together with the impact on the farmers’ livelihoods.

**Figure 4:**
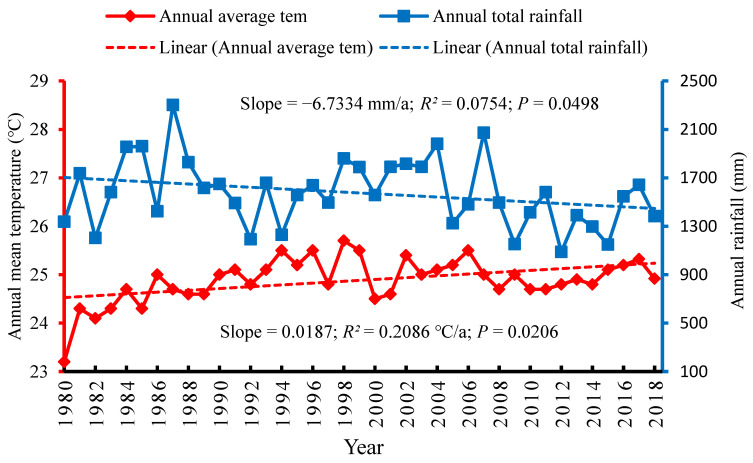
Climatic trend in the Tarai region of the trans-boundary KRB.

With respect to the Tarai region of the KRB, it was found that the mean annual temperature also showed an increasing trend by 0.0187 °C/a between 1980 and 2018. The increased rate of temperature for this region was significantly less than the mountain and hill regions of the basin. Furthermore, the rise in temperature trend in Tarai region also positively correlated with a *p* value of 0.0206 (Figure 4).

Moreover, the total annual precipitation between 1980 and 2018 in the Tarai region showed a clear decreasing trend of −6.7334 mm/a. The total annual precipitation for this region had a positive correlation with a *p* value of 0.0498 (Figure 4). The climatic trends in the Tarai region suggest that there were also significant impacts on the region from climate change, especially in the case of the decline in precipitation over the 38 year period, which significantly impacted the livelihoods of farmers and on the overall farming system.

**Figure 5:**
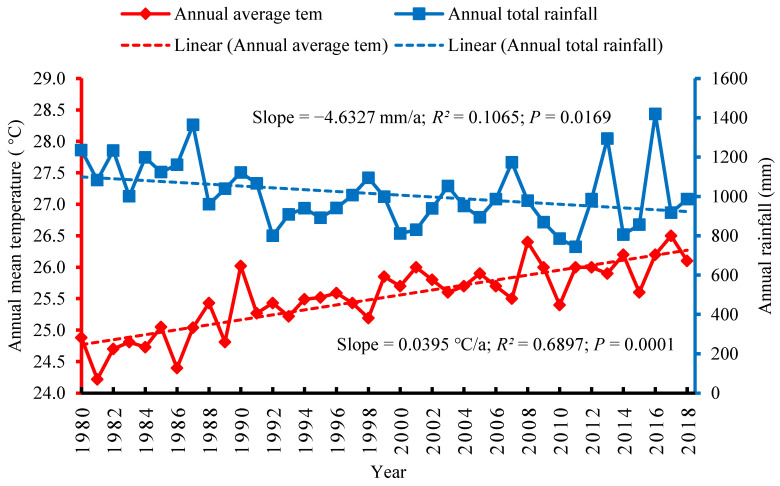
Climatic trend in the Gangetic plain of the trans-boundary KRB.

A climatic trend for the Gangetic plain located in the southern part of the trans-boundary KRB was noted, with the mean annual temperature increasing slightly with a rate of 0.0395 °C/a between 1980 and 2018. The increased rate of temperature in this region was significantly less than that for the mountain and hill regions of the basin, but was slightly higher than that for the Tarai region. The rising temperature trend of this region was correlated with the *p* value of 0.0001 (Figure 5). Furthermore, the total annual precipitation between 1980 and 2018 in the Gangetic plain changed slightly in this period, and decreased at a rate of −4.6327 mm/a. The total annual precipitation for this region correlated with the *p* value of 0.0169 (Figure 5). In terms of the overall climatic trend for the Gangetic plain, there was a slight increase in temperature as well as a slight decrease in precipitation, however, climate change did significantly impact the overall farming system and the livelihoods of the farmers in this region were adversely affected. An interesting feature of this region is low lying topography level, whereby the majority of the farmers in the region were impacted by climate change-induced flood disasters in the monsoon seasons and by drought events in the summer seasons.

### 3.2. Farmers’ Local Knowledge of Climate Change and Its Impact

#### 3.2.1. Observed Indicators of Climate Change

The farmers’ local knowledge is very important in the context of gaining information on what types of indicators in their locality are appropriate for climate change. This information on the respondent’s noted indicators of climate change for the four physiographic regions of the basin have been summarized in Table 2. The farmers identified six main indicators of climate change in their local regions. The specific indicators did vary according to the physiographic region. The changes of temperature and precipitation were especially recognized as indicators of climate change by farmers in the trans-boundary KRB with 90.9% and 90.5%, respectively. Farmers shared during KII and FGDs, and the majority of the respondents in all regions of the basin strongly perceived that temperature and precipitation were strong indicators of climate change. Furthermore, the increasing trend of temperature and the decreasing trend of precipitation perceived by the farmers were supported by the station-based climatic records between 1980 and 2018 in all regions.

**Figure 6:**
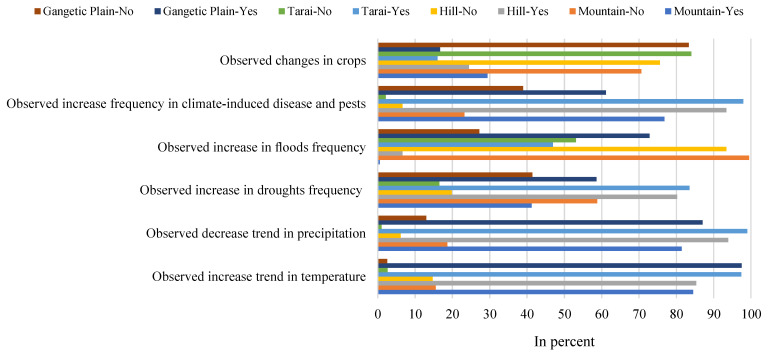
The observed indicators of climate change by local farmers in four physiographic regions of the trans-boundary KRB.

Furthermore, of the farmers surveyed, around 83.3% perceived that the increase in climate-induced diseases and pests was another strong indicator of climate change, whilst 66.3% perceived an increasing trend for drought to occur as a result of climate change. About 97.9% of farmers in the Tarai region remarked that the increase in climate-induced diseases and pests was one of the indictors of climate change, and this was also the perception for almost 61.1% of farmers in the Gangetic plain (Figure 6). Drought was highly perceived as an indicator of climate change for respondents in the Tarai region and hill regions of the basin (83.5% and 80.2%, respectively), but this was less so for the farmers in the mountain region (Table 2). Farmers shared that during KII and FGDs, the drought seriously impacted their overall agricultural activities and crop production in recent decades. The farmers also indicated that the changes in crop yield and flood frequency were also indicators of climate change. For instance, flood events, as indicators of climate change, were perceived mainly by the respondents’ of the Gangetic plain and the Tarai regions (72.8% and 46.9%, respectively), but much less so by farmers in the mountain and hill regions of the basin (Table 2). We noted that during KII and FGDs in the Gangetic plain, the livelihood of the farmers, especially in the monsoon season, was largely impacted by flood events in each year.

#### 3.2.2. Observed Impacts from Climate Change

**Figure 7:**
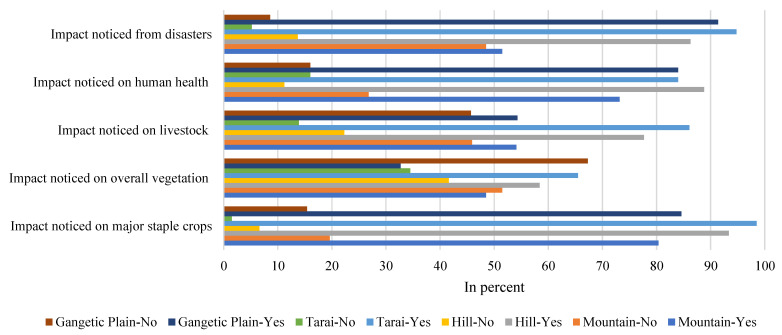
The observed impacts of climate change by local respondents’ in the various sectors in four physiographic regions of the trans-boundary KRB.

The respondents of the study perceived that their livelihoods were impacted by climate change. They especially pointed out the impact noticed on major staple crops, vegetation, human health, livestock, and impact of natural disasters (particularly in flood events in the Gangetic plain and the Tarai regions and hailstorms in most regions) (Table 3). The five sectors listed in Table 3 reflect the strongly perceived impacts of climate change in the basin. Furthermore, the impacts of climate on the different physiographic regions of the basin varied, for example, the farmers of the Tarai region and the Gangetic plain were mainly impacted by natural disasters, especially from floods; of course floods had less of a direct impact on the mountain and hill regions of the basin (Figure 7). The farmers of the basin mostly felt the adverse impacts of climate change on yields of staple crops and human health (89.4% and 82.5%, respectively). Furthermore, during the KII and FGDs, the farmers also pointed out that they perceived impacts from climate change on their staple crops, on human health, on their livestock, and also from climate-induced disasters. Full details of the farmers’ perceived impacts in various sectors for the different physiographic regions of the basin are summarized in Table 3.

### 3.3. The Characteristics of Agriculture and the Farming Households

#### 3.3.1. Characteristics of Farming Households

Information on farming households was collected based on the completed HSs for different regions of the trans-boundary KRB as summarized in Table 4. It was found that the heads of the household for the surveyed households were 81.8% male and 18.2% female. It was noted that the Gangetic plain region had the highest male head of household with a percentage of 90.7% (Table 4). The study found that 84.5% of respondents had both parents alive, 0.5% of respondents were divorced, and 15.0% of respondents were widowed. The marital status of the respondents varied according to the physiographic region. The highest literacy of the respondents was found for the hill region, which was a reflection of the better educational opportunities in this region. Of the surveyed households, the overall literacy level of respondents for the basin was 60.8% (Table 4).

The average age of the respondents in the basin was 55.9 years, with the Tarai region having the highest average age of 59.3 years while the Gangetic plain had the lowest average age of 52.2 years. Furthermore, the average family size in the basin was 5.8. The physical health of the respondents and the non-farming average monthly income per family including remittances varied according to the physiographic region. The respondents’ families were invariably engaged in non-farm activities as well as farming so that that the average monthly income from non-farming activities including remittances for each family was around 322,024 Nepalese rupees (NRS) (ca. 291 USD; based on the exchange rate of 110 NRS = 1 USD in 2018/2019). The highest average monthly income from non-farm activity was noted for the mountain region of the basin while the lowest monthly incomes occurred for those with relatively low non-farm incomes in the hill region of the basin (Table 4). Clearly, the household characteristics of the respondents for each region showed some variations and these features are summarized in Table 4. It was anticipated that the respondents’ overall household characteristics and their farming practices determined their perception and personal experiences of climate change and its overall impacts on their livelihoods.

#### 3.3.2. Agricultural Characteristics

The average land area of the farms of the surveyed persons in the basin was 0.77 ha and the farm area did vary with the physiographic region. The largest land area, 1.13 ha, was in the Gangetic plain while land area was lower in the hill region, being 0.53 ha (Table 5). The average number of livestock owned in the basin was 7.6. Throughout the study area, 92.2% of the land was privately owned and 7.8% of the land was rented and farmed under a land tenure system. With respect to land rental, the region with the highest percentage of rental land was the Gangetic plain region, in contrast to that of the mountain region, which was just 2.6%. Of the surveyed farmers, some 57.5% depended exclusively on natural rainfall for water supply while various means of irrigation were deployed by the remaining farmers. Almost 79.0% of the surveyed farmers of the Gangetic plain had access to irrigation and most of that was seasonal. The least irrigation facilities were found in the hill region of the basin (Table 5). The majority of respondents remarked that the cultivated land was relatively poor and/or infertile (71.4%). The availability of farm machinery, hybrid seeds, chemical fertilizers and pesticides was variable depending on the physiographic region and the variables by region are summarized in Table 5. It was anticipated that the agricultural characteristics experienced by the farmers might potentially influence their understanding of climate change and its overall impact.

## 4. Discussion and Policy Implication

This research revealed an increasing trend in temperature and a decreasing trend in precipitation between 1980 and 2018 from inspection of the observed climatic records of the trans-boundary KRB. A previous study of the KRB also examined the rainfall status for the past 34 years (1981–2015) and a decreasing trend was noted [41]. A recent study of eastern Nepal (central part of the trans-boundary KRB), clearly pointed out that the annual average rainfall decreased by −20 mm/a between 1997 and 2016 [42]. Furthermore, the trans-boundary KRB study found that the maximum temperature increased by 0.1 °C decade−1 between 1975 and 2010 [25]. The increasing trend of temperature resulted in high level shrinkage of snow and glaciers in the Himalaya region [43], which cause uncertainties in the review follow system, peoples’ livelihood, and irrigation system. A long-term study of rainfall in Bihar, India, which is located in the southern part of the KRB, mentioned that the annual average rate declined by −2.17 mm/a between 1901 and 2002 [44]. Most of the climatic studies of the KRB have examined the increasing trend of temperature and the deceasing trend of rainfall [26]. Thus, the findings of this study and previous studies clearly highlight the changing rates of temperature and rainfall in the trans-boundary KRB, and consequently, the clear impact on farming activities and the farmers’ livelihoods.

The surveyed farmers perceived that climate change had strongly impacted on their livelihoods, with a noticeable impact on the yields of staple crops due to the increasing frequency of drought events, the rising temperatures, and declining rates of rainfall. Studies in Nepal have pointed out that climate change has significantly impacted on the agricultural sector and production, especially staple crop production [45,46]. Furthermore, climate change induced diseases and pests have also negatively impacted on crop yields [47], thus contributing to a reduced agricultural output [48]. The information shared by farmers’ during the KII and the FGDs also revealed that climate change impacted farming activities and livelihoods. Due to such impacts, a shift in farming to non-farming activities has been reported in the KRB, and this resulted in the abandonment of farmland [49].

Water availability and irrigation facilities fulfill an essential role in sustaining the farming sector and maintaining overall agricultural production [50]. The farmers freely shared their views with us during the HS, KII, and FGD sessions, with the majority having deeply held views regarding precipitation-based farming where such a farming system and their livelihoods were highly impacted by drought events. Farmers also remarked that climate change induced diseases impacted their livestock and such cases had become more apparent in recent times. During the FGDs and KII, most of the farmers informed us that climate change had negative impacts on human health with climate change induced disease becoming more common in their communities in recent years. Farmers reported being more susceptible in recent years to diseases such as viral influenza, kala-zar, diarrhea, typhoid, and dengue fever, diseases that have also been the subject of human health studies linked to climate change [51,52].

The adverse effects of natural disasters such as flood events, hailstorms, and droughts were also considered to be more frequent in recent times. The farmers perceived that over the last two decades, natural disasters such as hailstorms negatively impacted crop production and yields. A crop water shortage index-based study of the trans-boundary KRB also clearly indicated an increasing drought status for the basin between 2000 and 2014 [19], and this adversely impacted the overall agricultural activity and the farmers’ livelihoods. Furthermore, during the FGDs, the farmers of the Gangetic plain expressed the view that rainfall had decreased over the past two decades, but with an increasing frequency of floods events, which caused damage to crops and property, thus negatively affecting livelihoods. The respondents in the mountain, hill, and Tarai regions perceived that overall rainfall was decreasing compared to previous years, however, seasonal and erratic heavy rainfall events were happening more often nowadays compared to previous years, and creating flood events, especially in the Tarai region and the Gangetic plain. Thus, the farmers of the Tarai region and Gangetic plain were more vulnerable to flood events. A recent study in the KRB mentions that due to such climate induced disasters, the farmers perceived a decline in their agricultural production, especially the production of staple crops [29]. The majority of the surveyed farmers in the four physiographic regions perceived significant negative impacts of climate change on their livelihoods and farming systems. Most of the indicators of climate change that were pointed out by the respondents’ impacted negatively on their livelihoods. Previous studies in the Himalayas region reported that farmers did recognize that increases in temperature and decreases in rainfall were strong indicators of climate change [53,54]. Similarly, the increasing trend of frequent flooding [55] as well as the increasing drought frequency were also perceived by farmers as indicators of climate change [16].

The farmers of all physiographic regions of the trans-boundary KRB clearly indicated that they were directly impacted by climate change with their livelihoods being more vulnerable to climate change. The reduction in farming income due to negative impacts of climate change arose from loss of farm production and medical expenses brought about due to climate change induced disease. Moreover, adverse effects included crop damage due to drought, hailstorms, and flood events, which posed an extra burden for farmers and made it more difficult to sustain their livelihoods. To minimize climate change related impacts, suitable adaptation strategies need to be developed. The concerned trans-boundary governments need to develop specific adaptation strategies to mitigate the impacts of climate change. The previous study indicated for focus on gender equity as well as to mitigate climate change impact [56]. Thus, the adaptation strategies need to be formulated at a local level based on consideration of the physiographic regions of the basin, practice based local knowledge of the farmers, gender equity, and ideas and proposals raised by the farmers’ during the KII and FGD sessions. The existing adaptation strategies need to be improved based on using the local knowledge of farmers and the local climatic status and geography. The results and discussions presented in this study and based on the farmers’ perceptions and station-based climatic records provide a clear description of the status and impact of climate change on the farmers’ livelihoods in the four physiographic regions of the trans-boundary Koshi Basin. To utilize these findings, it is recommended that suitable adaptation strategies and policies are formulated in favor of sustaining agriculture and improving the farmers’ livelihoods. Furthermore, it is recommended that local stakeholders, together with farmers, experts, government representatives, and accountable organizations, work collectively at the local level to devise appropriate solutions to minimize the impacts of climate change on the famers’ livelihoods and agricultural activity in general. The new adaptation strategies proposed include the introduction of infrastructure for irrigation systems, the development of crop seeds that are more tolerant to drought, pests, and disease tolerance, and the construction of local hospitals for the benefit of the farming communities.

## 5. Conclusions and Future Prospects

The climatic records for four physiographic regions of the trans-boundary KRB together with the famers’ local knowledge were used to examine climatic trends and overall impacts. Considering the high number of surveyed respondents as well as from the KII and the FGDs, it was noted that the trends of increasing temperature and decreasing rainfall were the main indicators of climate change in the four physiographic regions of the trans-boundary KRB. These findings were consistent with the recorded climate data for the regions between 1980 and 2018. An increasing frequency of drought was perceived as an indicator of climate change mainly for the hill region of the KRB and also for the Tarai region as well as the flooding frequency in the Tarai region and the Gangetic plain of the basin. The surveyed farmers remarked that, in the main, climate change impacted the yield of staple crops, human health, livestock, and vegetation; there were also impacts from natural disasters, and the rate of impact did vary according to the specific region in the basin.

The outcomes of this study are potentially useful for governmental bodies and policymakers who are involved in developing adaptation strategies to minimize the impact of climate change on the agricultural sector including the livelihood of farmers. The policy needs to cover and improve educational programs concerning climate change and focus on developing advancement of this sector (i.e., drought-tolerant crops, irrigation facilities, disease- and pest-resistant seeds, and more hospitals for the local communities). In this way, the farmers’ livelihoods can be improved, and the overall impacts of climate change can be reduced and in so doing, climate-resilient communities can develop and flourish. Furthermore, from the point of view of future prospects, the flooding events in the Tarai and Gangetic plain regions and drought events in most of the regions are the major concerns and challenges for the farmers, planners, and policymakers in the trans-boundary KRB. Thus, these well noticed issues are needed to focus on issue-based in-depth studies in the future as well as the appropriate actions highly needed to minimize impact from such climatic issues from the concerned authority and governmental bodies of the region. In addition, the experience of the farmers’ indigenous knowledge on climate change and its impact in these regions can potentially be useful for other similar topographies in the world where adaptation plans will be necessary.

## Figures and Tables

**Table 1:** Details of the sampled sites in the trans-boundary Koshi River Basin.

Site of Sampling	District	Physiographic Region	Number of HSs	Country
Nigale	Sindhupalchowk	Mt.	54	Nepal
Khawa	Dolakha	Mt.	54	Nepal
Chong	Sindhupalchowk	Mt.	54	Nepal
Taplejung	Taplejung	Mt.	32	Nepal
Chaukat	Kavrepalanchowk	H	57	Nepal
Karkigaun	Ramechhap	H	54	Nepal
Thakle	Okhaldhunga	H	54	Nepal
Yasok	Pachthar	H	32	Nepal
Chandrapur	Rautahat	T	54	Nepal
Kisannagar	Mahottari	T	54	Nepal
Ratanpur	Siraha	T	54	Nepal
Chakraghati	Sunsari	T	32	Nepal
Bharwari	Sitamarhi	GP	54	India
Benipur	Darbhanga	GP	54	India
Phulaut	Madhepura	GP	54	India

Note: Mt. refers to the mountain region, H for hill, T for Tarai and GP for Gangetic plain.

**Table 2:** The observed indicators of climate change by local farmers in four physiographic regions of the trans-boundary KRB.

Major Indicators	Basin Level Responses (%)		Physiographic Region Responses (%)							
			Mt.		H		T		GP	
	Y	N	Y	N	Y	N	Y	N	Y	N
Observed increase trend in temperature	679 (90.9)	68 (9.1)	84.5	15.5	85.3	14.7	97.4	2.6	97.5	2.5
Observed decrease trend in precipitation	676 (90.5)	71 (9.5)	81.4	18.6	93.9	6.1	99.0	1.0	87.0	13.0
Observed increase in droughts frequency	495 (66.3)	252 (33.7)	41.2	58.8	80.2	19.8	83.5	16.5	58.6	41.4
Observed increase in floods frequency	223 (29.9)	524 (70.1)	0.5	99.5	6.6	93.4	46.9	53.1	72.8	27.2
Observed increase frequency in climate-induced disease and pests	622 (83.3)	125 (16.7)	76.8	23.2	93.4	6.6	97.9	2.1	61.1	38.9
Observed changes in crops	163 (21.8)	584 (78.2)	29.4	70.6	24.4	75.6	16.0	84.0	16.7	83.3

Note: Total values were calculated based on 747 household surveys (HSs). Mt. refers to mountain region, H is for hill, T for Tarai, and GP for Gangetic plain. Furthermore, Y refers for Yes and N for No. Values by physiographic region were calculated based on 194 HSs for the mountain and Tarai regions, 197 HSs for the hill region, and 162 HSs for the Gangetic plain regions of the KRB. Sources: Field Surveys 2018 and 2019.

**Table 3:** The observed impacts of climate change by the local respondents’ in the various sectors for the trans-boundary KRB.

Observed Major Impacted Sectors	Basin Level Responses (%)		Physiographic Region Responses (%)							
			Mt.		H		T		GP	
	Y	N	Y	N	Y	N	Y	N	Y	N
Impact noticed on major staple crops	668 (89.4)	79 (10.6)	80.4	19.6	93.4	6.6	98.5	1.5	84.6	15.4
Impact noticed on overall vegetation	389 (52.1)	358 (47.9)	48.5	51.5	58.4	41.6	65.5	34.5	32.7	67.3
Impact noticed on livestock	513 (68.7)	234 (31.3)	54.1	45.9	77.7	22.3	86.1	13.9	54.3	45.7
Impact noticed on human health	616 (82.5)	131 (17.5)	73.2	26.8	88.8	11.2	84.0	16.0	84.0	16.0
Impact noticed from disasters	602 (80.6)	145 (19.4)	51.5	48.5	86.3	13.7	94.8	5.2	91.4	8.6

Note: Total values were calculated based on 747 HSs. Mt. refers to mountain region, H for hill, T for Tarai, and GP for Gangetic plain. Furthermore, Y refers for Yes and N for No. Values by physiographic region were calculated based on 194 HSs for the mountain and Tarai regions, 197 HSs for the hill region and 162 HSs for the Gangetic plain region of the KRB. Sources: Field Surveys 2018 and 2019.

**Table 4:** Attributes for heads of household for the farmers surveyed in the trans-boundary KRB.

Household Head Characteristics	Attribute	Total Number	%	By Physiographic Region (%)			
				Mountain	Hill	Tarai	Gangetic Plain
Gender of respondent	Male	611	81.8	83.0	76.1	78.9	90.7
	Female	136	18.2	17.0	23.9	21.1	9.3
Marital status	Both parents alive	631	84.5	86.1	82.7	77.3	93.2
	Divorced	4	0.5	1.0	0.0	0.5	0.6
	Widowed	112	15.0	12.9	17.3	22.2	6.2
Level of education	Illiterate	293	39.2	38.1	35.5	45.9	37.0
	Preschool/Informal	102	13.7	21.6	16.2	12.4	2.5
	Primary (1–5)	91	12.2	14.9	11.7	7.7	14.8
	Junior high school (6–10)	142	19.0	16.0	22.3	16.5	21.6
	Senior high school (11–12)	69	9.2	4.6	8.6	12.4	11.7
	Campus and above 12 class	50	6.7	4.6	5.6	5.2	12.3
Age of respondent (years)	Average age	55.9		54.5	56.9	59.3	52.2
Physical health	Good	438	58.6	64.9	56.3	67.0	43.8
	General	227	30.4	24.7	34.5	28.9	34.0
	Poor	65	8.7	8.2	7.1	3.6	17.3
	Very poor	17	2.3	2.1	2.0	0.5	4.9
Family size of respondent	Average family size	5.8		5.3	5.8	6.0	6.3
Average monthly income (NRS)	Non-farm family income including remittances	32,023.9		35,150.5	27,098.5	31,460.8	34,385.8

Note: Total values were calculated based on 747 HS. Values by physiographic region were calculated based on 194 HS for the mountain and Tarai regions, 197 for the hill region, and 162 for the Gangetic plain of the KRB, where NRS is Nepalese rupees, and Indian currency for lower part of the basin was converted into Nepalese currency to ensure the same metric for all regions. Sources: Field Surveys 2018 and 2019.

**Table 5:** Agricultural characteristics for the surveyed farmers in the trans-boundary KRB.

Agricultural Characteristics	Attribute Details	Total Number	%	By Physiographic Region (%)			
				Mountain	Hill	Tarai	Gangetic-Plain
Total land owned (hectare)	Average land area	0.77		0.79	0.53	0.71	1.13 ha
Total livestock owned	Average livestock size	7.6		10.1	8.2	7.1	4.2
Land tenure system	Land owned	689	92.2	97.4	96.4	95.4	77.2
	Land rented	58	7.8	2.6	3.6	4.6	22.8
Farming practices	Rain-fed cultivation	428	57.3	83.5	92.9	25.3	21.0
	Irrigated cultivation	319	42.7	16.5	7.1	74.7	79.0
Characteristics of soil	Fertile	214	28.6	29.4	20.3	33.0	32.7
	Normal or Infertile	533	71.4	70.6	79.7	67.0	67.3
Use of farm machinery	Yes	449	60.1	32.0	26.4	98.5	88.9
	No	298	39.9	68.0	73.6	1.5	11.1
Use of hybrid seeds	Yes	400	53.5	40.2	41.1	56.2	81.5
	No	347	46.5	59.8	58.9	43.8	18.5
Use of chemical fertilizers	Yes	631	84.5	58.8	86.8	96.4	98.1
	No	116	15.5	41.2	13.2	3.6	1.9
Use of pesticides	Yes	398	53.3	8.2	42.6	71.6	84.6
	No	349	46.7	91.8	57.4	28.4	15.4

Note: Total values were calculated based on 747 HS. Values by physiographic region were calculated based on 194 HS for the mountain and Tarai regions, 197 for the hill region, and 162 for the Gangetic plain of the KRB. Sources: Field Surveys 2018 and 2019.

## Data Availability

Not applicable.
